# Characterization of *Neospora Caninum* Microneme Protein 26 and Its Potential Use as a Diagnostic Marker for Neosporosis in Cattle

**DOI:** 10.3389/fvets.2020.00357

**Published:** 2020-07-17

**Authors:** Xianmei Wang, Xingju Song, Jing Yang, Qun Liu, Jing Liu

**Affiliations:** ^1^National Animal Protozoa Laboratory, China Agricultural University, Beijing, China; ^2^Key Laboratory of Animal Epidemiology of the Ministry of Agriculture, China Agricultural University, Beijing, China

**Keywords:** *Neospora caninum*, ESA, microneme protein 26, ELISA, cattle

## Abstract

The apicomplexan parasite *Neospora caninum* causes neosporosis, an illness that leads to abortion or stillbirth in cattle, causing massive economic losses to the livestock industry. Rapid and viable diagnosis is the premise of prevention and control for neosporosis. In this study, we screened a new microneme protein 26 (NcMIC26) through western blot and mass spectrometry identification from the excretory secretion antigen (ESA) of *N. caninum* tachyzoites. NcMIC26 is subcellularly localized to the microneme of parasites. NcMIC26 is a specific antigen of *N. caninum* and has no cross-reaction with *Toxoplasma gondii*. Therefore, NcMIC26 has the potential to be a candidate diagnostic antigen for neosporosis. To test this hypothesis, recombinant NcMIC26 (rNcMIC26) was expressed in *Escherichia coli* (*E. coli*), and an indirect ELISA for detecting anti-*N. caninum* antibodies in cattle was established. Compared with that of the indirect immunofluorescent antibody test (IFAT), the positive coincidence rate of the ELISA based on rNcMIC26 was 76.53% (75/98), which was higher than that of an ELISA based on rSRS2 (66.33%), and the negative coincidence rate was 84.62% (33/39). It is noteworthy that 30 positive samples confirmed by IFAT were consistent with the rNcMIC26 ELISA but were negative by the rNcSRS2 ELISA. Our research illustrated that NcMIC26 is a dependable diagnostic marker for the serodiagnosis of *N. caninum* infection in cattle and could be utilized as a supplementary antigen for missed detection by NcSRS2.

## Introduction

*Neospora caninum* is an obligate intracellular apicomplexan parasite that is the etiologic agent of neosporosis for a variety of animals, for which canids are the definitive hosts (1). The disease tends to be globally distributed and is most serious in cattle (2). Abortion is the main clinical symptom of infection, and neosporosis is one of the main causes of cattle abortion worldwide (3). Reproductive loss is the main clinical outcome of neosporosis in cattle and a major reason for the economic impact on the dairy and beef cattle trade (2). In the absence of an effective treatment or vaccine against bovine neosporosis, control of the disease depends on an accurate diagnosis of neosporosis-infected cattle for timely treatment or early elimination of livestock and other farm management measures.

The recombinant antigens used or validated for indirect ELISA are based on different biological function-associated antigens, including the surface antigens NcSAG1 (4), NcSRS2 (5), NcP40 (6), NcSAG4 (7), and rNcSRS9 (8); dense granule antigens NcGRA2 (9), NcGRA6 (10), and NcGRA7 (11); microneme antigen NcMIC10 (12); and other antigens, such as *Neospora* profilin (13). The NcSRS2 antigen is the most widely used and shows excellent diagnostic parameters (14). However, it is hard to examine the antibody response simultaneously against different antigens to which a host is differentially exposed depending on the stage (the acute or chronic stage) of *N. caninum* infection by using single antigens in ELISA. Similar to most other apicomplexan parasites, the process of invasion is necessary for *N. caninum* to survive and replicate within the host (15), and the proteins discharged by tachyzoites, known as the excretory secretion antigens (ESAs), are the most common targets of host immune reactions; recognizable proof of ESAs included in invasion may be valuable for revealing the critical target for and the prevailing antigen of *N. caninum* (16). In the present study, we screened a microneme protein 26 (NcMIC26) from *N. caninum* ESAs. The localization of NcMIC26 is in the microneme of parasites, and it partially colocalizes with NcMIC4. Recombinant NcMIC26 was expressed in *Escherichia coli*; a reliable, sensitive, and specific diagnostic test based on recombinant NcMIC26 was developed; and its diagnostic potential in an ELISA was evaluated.

## Materials and Methods

### Parasites and Cell Cultures

*N. caninum* Nc-1 strain tachyzoites were propagated in African green monkey kidney (Vero) cells cultured in Dulbecco's modified Eagle's medium (DMEM) (M&C, China) containing 25 mM glucose and 4 mM glutamine and supplemented with 2% fetal bovine serum (FBS, Gibco, USA). Cells were incubated at 37°C with 5% CO_2_ in a humidified incubator.

### Preparation of *N. caninum* Tachyzoite ESA and Soluble *N. caninum* Lysate Antigen

To find a new *N. caninum* diagnostic antigen, we employed mass spectrometry-based proteomics to identify proteins present in the *N. caninum* tachyzoite using two different approaches. The first approach was identifying the proteins present in the tachyzoite-secreted fraction (ESA). ESA were obtained according to a method involving *Toxoplasma gondii* that was previously described (17). Briefly, the tachyzoites were harvested from Vero cell cultures. Twenty-seven-gauge needles were used to disrupt the cells, and lysates were filtered through a 5-μm syringe filter. The purified tachyzoites were washed three times in DMEM by centrifugation at 900 × g for 10 min. The freshly purified tachyzoites were incubated (5 × 10^7^ parasites/mL) in a serum-free medium (DMEM) at 37°C for 3 h and cooled for 10 min on ice. The supernatant separated from the parasites and containing ESA was collected by centrifugation at 20,000 × g for 10 min at 4°C. The parasites were lysed using a RIPA buffer (Beyotime, China) supplemented with a cocktail of protease inhibitors (Sigma, USA).

The second approach was to identify the secreted proteins in the culture medium of intracellular tachyzoite cultures. Nc1 tachyzoites were inoculated in Vero cells. The medium was discarded after 3 days before its egress and washed with PBS three times, and then a serum-free DMEM was cultured at 37°C for 24 h. A dialysis bag (Harveybio, China) was used to concentrate the collected secreted proteins from intracellular culture. The medium collected from the Vero cell culture served as the control.

### Identification of *N. caninum*-Specific Antigen From Tachyzoite ESA

The polyclonal antiserum against *N. caninum* or *T. gondii* was generated in mice or rabbits using tachyzoites lysate antigen as described previously (18). Six- to eight-week-old female BALB/c mice and 2-month-old rabbits were purchased from the Academy of Military Medical Sciences Laboratory Animal Center (Beijing, China). Mice and rabbits were immunized subcutaneously every 2 weeks with 100 μg (mice) or 2 mg/kg (rabbits) tachyzoites lysate antigen in an equal volume of Freund's complete adjuvant (Sigma, USA) for the first injection and 50 μg (mice) or 1 mg/kg (rabbits) for the second and third injections. The anti-*N. caninum* or anti-*T. gondii* sera were collected 10 days after the final immunization. Animal experiments were conducted according to the institutional guidelines for animal ethics.

SDS-PAGE and western blot were used to identify the collected *N. caninum* proteins, which were performed as previously described (6). The *N. caninum* tachyzoite ESA and lysate samples were loaded into a 12% SDS-PAGE gel with equal loads. Separated protein bands were visualized in gels by silver staining according to the manufacturer's protocol for the Silver Stain Kit (Beyotime Biotechnology Co., Ltd., China) or transferred to polyvinylidene fluoride (PVDF) membranes (Millipore, MA, USA) together with a visible prestained protein marker (TransGen Biotech Co., Ltd., China) after electrophoresis (Bio-Rad). The membranes were blocked with 5% (w/v) skim milk (BD Difco, USA) in PBS for 1 h at 37°C, rinsed with a washing buffer, and incubated with a mouse polyclonal antiserum against *N. caninum* (1:400) or a rabbit polyclonal antiserum against *T. gondii* (1:400) for 1 h at 37°C. The blots were washed five times with PBST (1% Tween-20), followed by incubation with horseradish peroxidase (HRP)-labeled goat antimouse IgG (H + L) (1:5,000, Sigma, USA) or HRP-labeled goat antirabbit IgG (1:10,000, Sigma, USA). Finally, enhanced chemiluminescence reagents (CoWin Biotech Co., Ltd., China) were used to observe the reaction bands after 5 s of exposure time.

According to the western blot results, the corresponding specific bands (realized by anti-*N. caninum* but not anti-*T. gondii* antibodies) in SDS-PAGE protein strips were cut out for mass spectrometry (MS) and protein identification (Beijing Protein Innovation Co., Ltd., China) to obtain the corresponding peptide information and determine the *N. caninum*-specific antigen. Analysis of the DNA and protein sequences was performed using The Toxoplasma Genomics Resource (ToxoDB) website (https://toxodb.org/toxo/) and the National Center for Biotechnology Information (NCBI) website (https://www.ncbi.nlm.nih.gov/). Conserved domains are available on the NCBI Conserved Domain Search (https://www.ncbi.nlm.nih.gov/Structure/cdd/wrpsb.cgi).

### Gene Cloning, Recombinant Protein Expression, and Purification

Total RNA was extracted from purified *N. caninum* tachyzoites, reverse-transcribed (TransGen Biotech Co., Ltd., China), and used as a template for PCR. Primers were designed for the region 271–1,014 bp of the NcMIC26 RNA sequence (ToxoDB, NCLIV_033690), with low similarity with the homologous gene in *Toxoplasma gondii*. The primer sequences were as follows—forward primer: 5′-AGCAAATGGGTCGC@@UGGATCCLINE@@GTTCTGGATTTCATAGACTTGG-3′, containing a *Bam*HI site, and reverse primer: 5′-TCGAGTGCGGCCGC@@UAAGCTTLINE@@CGAAGTCCATTCGCCCCACGTT-3′, containing a *Hind*III site. The truncated NcMIC26 gene was amplified with 2 × ExTaq Mix polymerase (TransGen Biotech Co., Ltd., China), and PCR was performed using the following procedure: 95°C for 5 min; followed by 35 cycles at 95°C for 30 s, 57°C for 1 min, and 72°C for 1 min; and extension at 72°C for 10 min.

The resultant PCR products were ligated into the pET-28a expression vector backbone after *Bam*HI and *Hind*III double enzymatic digestion. The insert sequences were sequenced and aligned to the NcMIC26 gene sequence reported previously (19). The recombinant plasmid was named pET-28a-NcMIC26.

The recombinant plasmid was transformed into *Escherichia coli* Transetta (DE3). Expressed as a His tag fusion protein following induction with 0.8 mM IPTG for 6 h at 37°C, rNcMIC26 was purified by column chromatography using Ni-NTA Superflow columns and stored at −80°C until use.

### NcMIC26 Subcellular Localization

NcMIC26 endogenous epitope tags were generated as described previously (20). Briefly, the plasmid pNc_Cas9CRISPR::sgNcMIC26 and the plasmid pLIC-HA-DHFR-NcMIC26 (a template of homologous repair amplicon) were constructed, and the primers are listed in Table S1. Then, the parental Nc1 strain was cotransfected with the plasmid pNc_Cas9CRISPR::sgNcMIC26, and a linearized homologous repair was completed. The transgenic parasites were grown under pyrimethamine (1 μM) selection pressure to the third generation and then screened to confirm the purity of the selected strains. The selected strain was named NcMIC26-HA.

IFAT was used for NcMIC26 subcellular localization in parasites as previously described (20). Briefly, 1 × 10^5^ *N. caninum* tachyzoites were seeded onto HFFs that were already arranged on glass coverslips in 12-well-plates (Corning costar, USA). Infected cells were incubated at 37°C with 5% CO_2_ for 30 h, fixed for 30 min in 4% formaldehyde, permeabilized with 0.25% Triton X-100 for 15 min, and then blocked with 3% bovine serum albumin (BSA) for 30 min. Subsequently, the cells were incubated with a mouse anti-HA monoclonal antibody (1:50, Sigma-Aldrich) and a rabbit antiNcSRS2 polyclonal antibody [1:500, (20)], the primary antibodies were detected with FITC-conjugated goat-anti mouse IgG (H + L) (1:100, Sigma, USA) and Cy3-conjugated goat-anti rabbit IgG (H + L) (1:100, Sigma, USA), respectively, and the nuclear DNA was stained with DAPI (1:200, Sigma, USA). Finally, a Leica confocal microscope system (Leica, TCS SP52, Germany) was used to obtain images.

### Specificity Analysis

The expression and purity of recombinant protein were analyzed by SDS-PAGE electrophoresis and visualized by Coomassie blue staining. Besides, SDS-PAGE and western blots were used to confirm the reactogenicity and antigenic specificity of *N. caninum*. Western blots were followed as described above; rNcMIC26 was subjected to electrophoresis and transferred electrophoretically onto a PVDF membrane. After blocking, the blots were incubated with mouse polyclonal antiserum against *N. caninum* (1:400), mouse *N. caninum*-negative serum (1:400), mouse polyclonal antiserum against *T. gondii* (1:400), and mouse anti-His monoclonal antibody (1:500) for 1 h at 37°C. After washing, the cells were incubated with a horseradish peroxidase (HRP)-labeled goat anti-mouse IgG (H + L) secondary antibody (1:5,000).

### Indirect ELISA

Indirect ELISA tests based on the purified recombinant protein NcMIC26 were developed as previously described (21). Optimal dilutions of the antigen and bovine sera were determined by checkerboard titration. *N. caninum*-positive and negative sera samples were employed to each assay. We diluted the His-fused rNcMIC26 in a coating buffer (0.05 M carbonate–bicarbonate buffer, pH 9.6) to a final concentration of 1 μg/well, added it to 96-well flat-bottom plates (Guangzhou Jet Bio-filtration Co., Ltd., China), and incubated it at 37°C for 1 h and then at 4°C overnight. After four washes with a washing buffer (PBS containing 0.1% Tween 20), the plates were blocked with a blocking buffer (PBS containing 5% horse serum) at 37°C for 1 h. The plates were washed four times, the cattle sera were diluted in a diluent solution (PBS containing 2% horse serum, 1:200), and 100 μl was added to each of the duplicate wells of the ELISA plate and incubated for 1 h at 37°C. The plates were rinsed as before and incubated with the HRP-conjugated goat anti-bovine IgG antibody (Southern Biotechnology Associates, Inc., USA) diluted in a diluent solution (1:25,000) at 100 μl/well for 0.5 h at 37°C. Finally, the plate was rinsed, and bound antibodies were detected by incubation with a 100 μl/well tetramethylbenzidine (TMB) substrate (M&C Gene Technology Co., Ltd., China) and color rendering at room temperature for 10 min. The reaction was stopped with a stop solution (2 M sulfuric acid, 50 μl/well), and the absorbance was measured at 450/630 nm in an ELISA plate reader (Bio-Rad). Every experiment was repeated three times. The cutoff point was determined as the mean OD_450/630nm_ for *N. caninum*-negative sera kept in our laboratory (*n* = 30) plus three standard deviations. The positive antibody of *N. caninum* was found in samples whose OD value ≥ cutoff point, and the sample OD value < cutoff point was negative for *N. caninum*.

### Data Analysis

The sera used in this study were as follows: gold-standard panel sera consisting of IFAT-defined negative sera (*n* = 39) and IFAT-defined positive sera (*n* = 98) from cattle. The number of positive or negative sera samples of the two diagnostic methods was counted manually. Statistical analysis of the data was performed using SPSS 22. Kappa coefficient was used to evaluate the level of agreement between ELISA methods (based on NcSRS2 or NcMIC26) and the gold standard (IFAT). The specificity and sensitivity of detecting *N. caninum* serum antibodies by ELISA were determined using the following formulas: sensitivity = (number of ELISA test-positive sera/number of IFAT test-positive sera) × 100%; specificity = number of ELISA test-negative sera/number of IFAT test-negative sera) × 100%). Indirect ELISA methods based on the rNcMIC26 and rNcSRS2 (5) antigens were used to detect these sera, and the relevant data were compared and analyzed.

### Ethics Statement

The experiments with animals in this study were performed strictly according to the recommendations of the Guide for the Care and Use of Laboratory Animals of the Ministry of Science and Technology of China and approved by the Institutional Animal Care and Use Committee of China Agricultural University (under the certificate of Beijing Laboratory Animal employee ID: CAU20161210-2).

## Results

### Identified NcMIC26 From ESA of *N. caninum*

**Figure 1:**
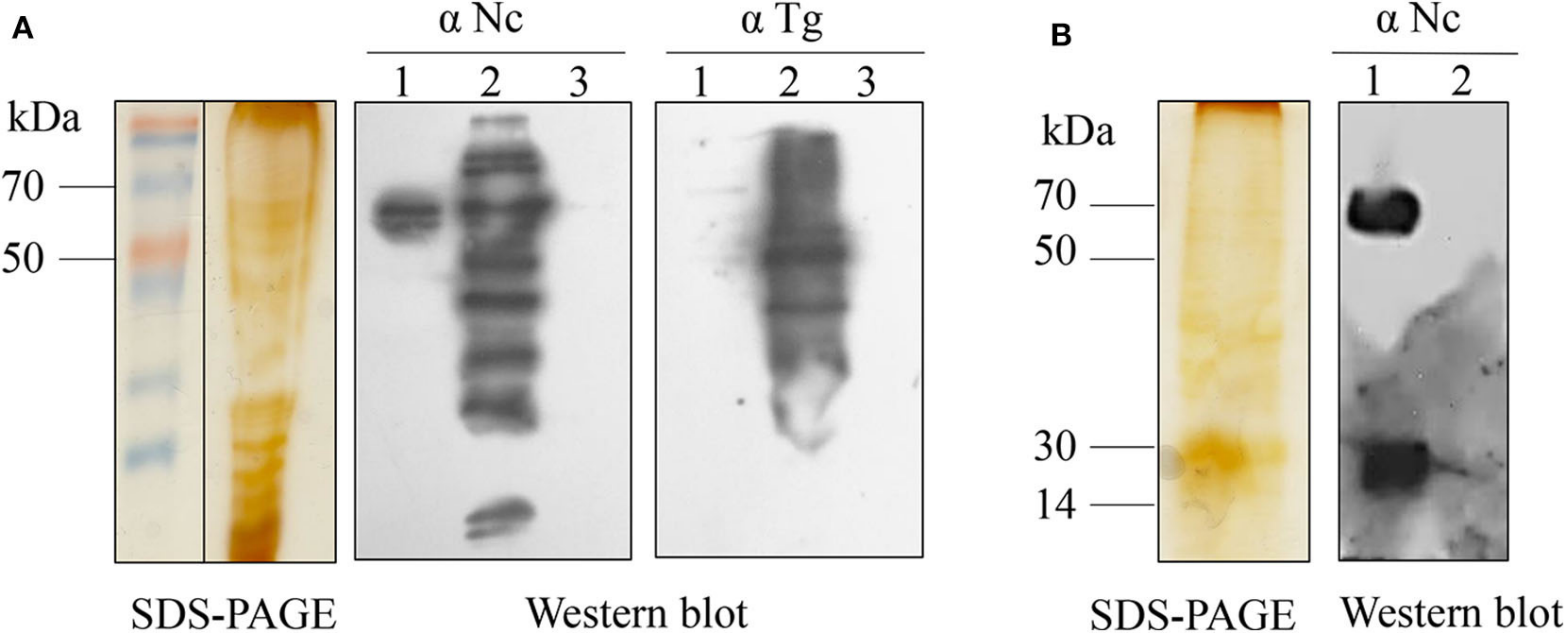
SDS-PAGE and western blot analyses of *N. caninum* ESAs. **(A)** SDS-PAGE and western blot analyses of *N. caninum* tachyzoite-secreted fraction ESAs. Lane 1, *N. caninum* tachyzoite-secreted fraction ESA; lane 2, *N. caninum* tachyzoite lysate; lane 3, DMEM control. Western blot using anti-*N. caninum* serum (1:400) or anti-*T. gondii* serum (1:400). **(B)** SDS-PAGE and western blot analyses of *N. caninum* tachyzoite-secreted protein for intracellular culture. Lane 1, *N. caninum* tachyzoite secreted protein from intracellular culture; lane 2, Vero cell control. Western blot using anti-*N. caninum* serum (1:400).

The collected ESA proteins were separated by SDS-PAGE, and western blot was used to identify antigen reactivity and specificity (Figure 1). Three specific protein bands (50–70 kDa from Figure 1A; 14–30, 50–70 kDa from Figure 1B) of *N. caninum* showed no reaction with *T. gondii*-positive serum. The tryptic peptides were analyzed by LC-MS/MS, corresponding to a total of 121 proteins after the appropriate cutoff filters were applied to the results (Table 1). First, we excluded proteins that were inconsistent with their corresponding band mass. Then, we mainly screened for proteins related to the three major secretory organelles and, eventually, determined NcMIC26 to be a candidate antigen of *N. caninum*, which was present in the tachyzoite-secreted fraction ESA (50–70 kDa from Figure 1A).

**Table 1:** *N. caninum* antigens from the tachyzoite ESA and tachyzoite-secreted proteins by LC-MS/MS.

**Gene ID**	**Weight**	**Name**	**Orthologous organism**	**Orthologous gene**
NCLIV_021050	93,650	Unnamed protein product	*Toxoplasma gondii ME49*	Subtilisin SUB1
NCLIV_001970	67,066	Predicted rhoptry protein kinase (ROPK)	*Toxoplasma gondii ME49*	Rhoptry protein ROP7
		Predicted rhoptry kinase, subfamily ROP24	*Toxoplasma gondii ME49*	Rhoptry protein ROP4
NCLIV_012920	43,208	Predicted rhoptry kinase, subfamily ROP23	*Toxoplasma gondii GT1*	Rhoptry family protein ROP40
NCLIV_060730	61,541	Predicted rhoptry protein kinase (ROPK)	*Hammondia hammondi strain H.H.34*	Rhoptry protein ROP5
		Predicted rhoptry kinase, subfamily ROP32	*Toxoplasma gondii ME49*	Rhoptry protein ROP5
NCLIV_043270	50,154	Putative microneme protein MIC1	*Toxoplasma gondii ME49*	Microneme protein MIC1
NCLIV_011730	59,828	Predicted rhoptry pseudokinase, subfamily ROP26	*Toxoplasma gondii GT1*	Rhoptry kinase family protein ROP26
			*Hammondia hammondi strain H.H.34*	Rhoptry kinase family protein ROP26
			*Sarcocystis neurona SN3*	Rhoptry kinase family protein ROP26
NCLIV_031550	48,172	Predicted rhoptry kinase	*Cystoisospora suis strain Wien I*	Rhoptry kinase family protein rop37
NCLIV_028170	63,997	ROP40	*Toxoplasma gondii GT1*	Rhoptry kinase family protein ROP20
NCLIV_045870	22,773	GRA3	*Toxoplasma gondii ME49*	Dense granule protein GRA3
NCLIV_036400	19,849	GRA1	*Toxoplasma gondii ME49*	Dense granule protein GRA1
NCLIV_033690	82,521	MIC26	*Cystoisospora suis strain Wien I*	Microneme protein
			*Toxoplasma gondii GT1*	Microneme protein MIC2
NCLIV_002940	66,537	Putative microneme protein MIC4	*Toxoplasma gondii ME49*	Microneme protein MIC4
NCLIV_028680	64,290	Putative apical membrane antigen 1	*Toxoplasma gondii GT1*	Apical membrane antigen AMA1

The gene sequence and the amino acid sequence of *N. caninum* NcMIC26 were obtained from ToxoDB (http://toxodb.org/toxo/; Gene ID: NCLIV_033690). The full-length NcMIC26 protein is composed of 756 amino acids, and a hydrophobic region at the N-terminus has characteristics of a signal peptide (1–33 amino acid). The mature protein has a predicted molecular weight of 80 kDa. Two putative transmembrane spanning helixes were found between amino acid residues 12–34 and 687–709, near the N-terminus and C-terminus of NcMIC26, respectively. In addition, the protein contains a Von Willebrand factor type A (vWA) domain (67–248) and five thrombospondin type 1 (TSP-1) repeat regions (261–325, 330–388, 394–449, 454–510, and 516–575). The NcMIC26 amino acid sequence aligned to other similar Apicomplexa proteins; the deduced NcMIC26 amino acid sequence was 45% identical to that of *N. canimum* MIC2 (NCLIV_022970), 44% identical to that of *T. gondii* MIC2 (TGGT1_201780), 59% identical to that of *Cystoisospora suis* microneme protein (CSUI_022748), and 52.76% identical to that of *Sarcocystis neurona SO SN1* syntenic protein (SRCN_7088).

### Recombinant NcMIC26 Had No Cross-Reaction With *T. gondii-*Positive Serum

**Figure 2:**
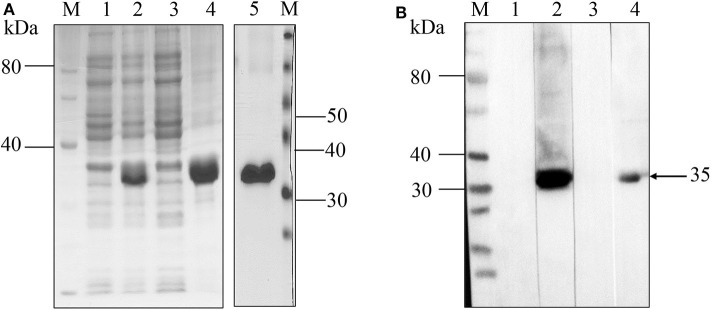
SDS-PAGE and western blot analyses of recombinant NcMIC26 protein. **(A)** Expression and purification of rNcMIC26. Recombinant protein expression patterns were analyzed by SDS-PAGE and visualized by Coomassie blue staining. M: protein marker; lane 1, uninduced protein; lane 2, induced protein; lane 3, supernatant; lane 4: inclusion bodies; lane 5, recombinant protein purified by Ni-NTA Superflow columns. **(B)** Western blotting was used to confirm the reactogenicity and antigenic specificity of *N. caninum*. M: protein marker; lanes 1–4 loading sample: purified rNcMIC26. The incubated antibodies were as follows: lane 1, non-infected mouse serum (1:400); lane 2, mouse *N. caninum*-positive serum (1:400); lane 3, mouse *T. gondii*-positive serum (1:400); lane 4, mouse anti-His monoclonal antibody (1:500); and HRP-labeled goat anti-mouse IgG (H + L) secondary antibody (1:5,000).

The coding sequence of the 744 bp truncated NcMIC26 gene encoding a target protein of 248 amino acids was inserted into the bacterial expression vector pET-28a and expressed as a His fusion protein in *E. coli*, with a predicted molecular mass of 35 kDa. The recombinant NcMIC26 was expressed mainly as inclusion bodies (Figure 2A). The molecular mass of the purified recombinant protein was 35 kDa, as expected. Western blot analysis showed that rNcMIC26 reacted strongly with *N. caninum*-positive serum and had no reaction with *T. gondii*-positive serum (Figure 2B). This result indicates that the recombinant protein rNcMIC26 has strong reactivity and that rNcMIC26 does not react with *T. gondii*-positive serum, possibly making it a candidate diagnostic antigen for neosporosis.

### NcMIC26 Localized on Micronemes

**Figure 3:**
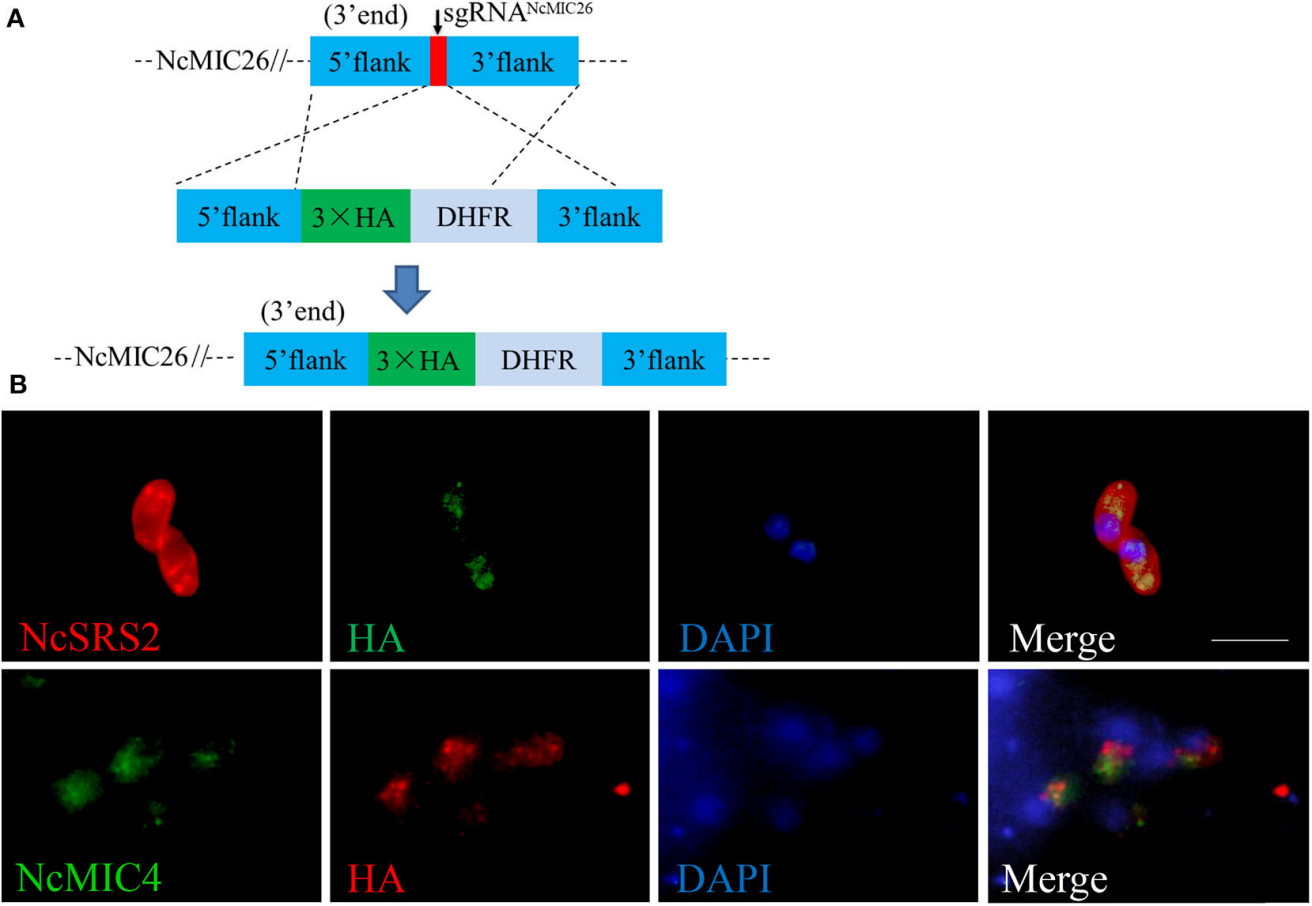
Characterization of NcMIC26 in *N. caninum*. **(A)** Schematic diagram showing NcMIC26 with an HA tag at its endogenous locus. **(B)** Localization of NcMIC26. NcMIC26, stained with mouse anti-HA antibody, was distributed in the microneme of NcMIC26-HA parasites. NcSRS2 was used as a marker to indicate the outlines of parasites; NcMIC4 was used as a marker to indicate the microneme of parasites. Visualized with Cy3-conjugated goat-anti-rabbit IgG (H + L) and FITC-conjugated goat-antimouse IgG (H + L), and nuclear DNA was stained with DAPI (blue). Scale bar, 5 μm.

To localize NcMIC26, NcMIC26 was fused with a triple hemagglutinin (3 × HA) epitope tag in the C-terminus by single homologous recombination (Figure 3A). NcMIC26 subcellular localization was visualized by immunofluorescent staining using a mouse anti-HA monoclonal antibody (1:50, Sigma-Aldrich) and rabbit anti-NcSRS2 polyclonal antibody (1:500) (20).

The anti-HA-labeled parasites showed that NcMIC26 was distributed in the micronemes of parasites (Figure 3B) and partially colocalized with another microneme marker, NcMIC4 (22), suggesting that NcMIC26 is a microneme protein in *N. caninum*.

### Diagnosis of *N. caninum* Infection in Cattle by ELISA With rNcMIC26

Ninety-eight samples of *N. caninum*-positive and 39 samples of *N. caninum*-negative bovine serum were defined by IFAT and evaluated by indirect ELISA based on the rNcMIC26 and rSRS2 antigens; OD values were listed in Table S2. The sensitivity and specificity of the ELISA were evaluated. The results are shown in Table 2. Compared with that of IFAT, the positive coincidence rate (sensitivity) of the ELISA based on rNcMIC26 (cutoff = 0.150) was 76.53% (75/98), and the negative coincidence rate (specificity) was 84.62% (33/39), while those of the ELISA based on rNcSRS2 (cutoff = 0.178) were 66.33% (65/98) and 92.31% (36/39), respectively. The sensitivity of the ELISA based on NcMIC26 was better than that of the ELISA based on NcSRS2. In addition, compared with NcSRS2 (kappa = 0.476), the NcMIC26-based ELISA test (kappa = 0.540) was more in agreement with the IFAT test through the calculation of the kappa value.

**Table 2:** Comparison between the ELISA based on rNcMIC26t or rSRS2 and IFAT coincidence rate.

**Antigen**	**Positive coincidence rate**	**Negative coincidence rate**	**Kappa value**
NcSRS2	66.33%(65/98)	92.31%(36/39)	0.476
NcMIC26	76.53%(75/98)	84.62%(33/39)	0.540

Afterward, a total of 137 serum samples were analyzed statistically. As shown in Table 3, the diagnosis results for bovine serum were not consistent. It is interesting to note that of the 98 *N. caninum*-positive bovine serum samples, 17 samples were confirmed as positive only by the ELISA based on rNcSRS2, while they were confirmed as negative by the ELISA based on rNcMIC26, and 30 samples were confirmed as positive by the ELISA based on rNcMIC26, while they were confirmed as negative by the ELISA based on rNcSRS2.

**Table 3:** Comparison between the ELISA based on rNcMIC26t and rSRS2 for the diagnosis of neosporosis in cattle.

**rNcSRS2**	**rNcMIC26**		
	**Positive No**.	**Negative No**.	**Total**
Positive No.	51	17	68
Negative No.	30	39	69
Total	81	56	137

## Discussion

*N. caninum* is a recently recognized protozoan parasite. Until 1988 (23), it was misdiagnosed as *T. gondii*. It is structurally, antigenically, and molecularly related to *T. gondii*, but these organisms are biologically distinct. One of the parameters to be evaluated is diagnostic specificity, that is, the proportion of test negatives among all animals that are true negative. Cross-reactivities among *N. caninum* and *T. gondii* have been reported (24). An increasing number of proteins have been identified as cross-antigens between the two closely related parasites, for example, MIC3 and AMA1 (25), affecting the diagnostic specificity of serological tests. Therefore, it is urgent to screen new dominant antigens with improved diagnostic abilities and to establish more sensitive and specific serological diagnostic methods.

In this study, a new diagnostic antigen, NcMIC26, from *N. caninum* tachyzoite ESA was screened. This antigen is composed of a Von Willebrand figure type A (vWA) domain and five thrombospondin type 1 (TSP-1) repeat regions. Since its identification, the vWA domain has drawn incredible interest because of its far-reaching effects and its association in a wide assortment of vital cellular functions. In *T. gondii*, the VWA domain likely intervenes in the protein–protein interaction of these proteins with their binding partners, which plays a pivotal part in cell adhesion and intrusion by interceding gliding motility (26). Within the TSP repeat region, several motifs are present that have been implicated in cell binding (27). For most MICs, secretion is started *in vitro* before parasites initiate egress from the host cells. Sera from actually infected cattle recognized an overwhelming protein band with an atomic mass indistinguishable from that of NcMIC26 in *N. caninum* ESA. However, it is possible that there are some proteins from the lysis of parasites in the detected proteins. To avoid this, we have selected the proteins from the three major secretory organelles (microneme, rhoptry, and dense granule) and detected its secretion ability in the following screening process. Finally, NcMIC26, which contains signal peptide and a transmembrane region, was confirmed as a secreted protein by secretion assays (data were not shown). In addition to the affirmation that the bovine antiserum recognized rNcMIC26, the results of the current study suggest that the bovine antisera against the whole parasite recognized NcMIC26 as an immunodominant antigen.

To assess whether recombinant NcMIC26 can be an appropriate antigen for the diagnosis of *N. caninum* disease in cattle, purified recombinant NcMIC26 was assessed in an ELISA. IFAT and the rNcSRS2-based ELISA were used as the comparison test. IFATs are based on intaglio tachyzoites and are respected, among others, as reference serological tests (“gold-standard tests”) (28). NcSRS2 is an immunodominant surface protein that is displayed within the bradyzoites and tachyzoites of *N. caninum* (29), and empowers specific serological diagnosis of neosporosis. These results demonstrated that the recombinant NcMIC26 expressed in *E. coli* ought to be a valuable diagnostic reagent for the detection of antibodies to *N. caninum* in cattle. Moreover, our detection information reflects a substantial discrepancy in the overall serum assessment determined by ELISA based on NcSRS2 or NcMIC26. The sensitivity of the ELISA based on NcMIC26 was better than that of the ELISA based on NcSRS2, and a considerable portion of *N. caninum*-positive serum can recognize only one antigen (only NcSRS2 or only NcMIC26). Using single antigens in ELISAs is insufficient to examine simultaneously the antibody response against different antigens to which a host is differentially exposed depending on the stage of infection (i.e., the acute or chronic stage). Our data indicated that the ELISA test utilizing NcMIC26 could be used as a supplementary antigen for missed detection by NcSRS2 to improve *N. caninum* diagnosis, while further study should focus on using both antigens in the same ELISA to prove the advantage. On the other hand, compared with that of IFAT, the positive coincidence rate of the ELISA based on rNcSRS2 (66.33%) in our research may be lower than that in other investigations (30). Considering that our serum samples are from cattle that were naturally infected with *N. caninum*, the stage of infection with *N. caninum* was inconsistent, and the antibody titers may be diverse.

## Conclusion

In this study, we screened a new microneme protein 26 (NcMIC26) from the excretory secretion antigen (ESA) of *N. caninum* tachyzoites. This study characterized NcMIC26 as an effective microneme protein that is recognized by the sera of *N. caninum*-infected animal hosts. The ELISA specific to NcMIC26 established in this study can aid in a more conclusive determination of *N. caninum* infection in cattle. Ensuing studies will be vital to extending the detection affectability of the assay, and more importantly, it can also be utilized as a supplementary antigen for missed discovery by SRS2. The combination of these two antigens may be considered to obtain more accurate detection information in clinical diagnosis.

## Data Availability Statement

All datasets generated for this study are included in the article/Supplementary Material.

## Author Contributions

JL conceived the project. XW performed the experiments and drafted the manuscript. JL and QL participated in the design of the study and helped to draft the manuscript. XW, JY, and XS participated in the interpretation of the data. All authors read and approved the final manuscript. All authors contributed to the article and approved the submitted version.

## Conflict of Interest

The authors declare that the research was conducted in the absence of any commercial or financial relationships that could be construed as a potential conflict of interest.

## Supplementary Material

Table S1 Primers of constructed plasmids pNc_Cas9CRISPR::sgNcMIC26 and pLIC-HA-DHFR-NcMIC26.

Table S2 The data of tested samples for indirect ELISA based on the rNcMIC26 and rNcSRS2.

## Abbreviations

*N. caninum*: *Neospora caninum*
*T. gondii*: *Toxoplasma gondii*
*E. coli*: *Escherichia coli*
ESA: excretory secretion antigen
SDS-PAGE: sodium dodecyl sulfate-polyacrylamide gel electrophoresis
LC-MS/MS: liquid chromatography mass spectrometer
WB: western blotting
ELISA: enzyme-linked immunosorbent assay
PCR: polymerase chain reaction
IFAT: indirect immunofluorescent antibody test
IPTG: isopropyl-β-d-thiogalactoside
MIC: microneme protein
SRS: surface antigen related protein
SAG: surface antigen
GRA: dense granule protein
DHFR: dihydrofolatereductase
HRP: horseradish peroxidase
FITC: fluorescein isothiocyanate.
