# Supplementary material for: Characterization of Neospora Caninum Microneme Protein 26 and Its Potential Use as a Diagnostic Marker for Neosporosis in Cattle
Source: Front Vet Sci. 2020 Jul 17;7:357. doi: 10.3389/fvets.2020.00357 (PMC7380076; doi:10.3389/fvets.2020.00357)
Supplement: Table S1 — Primers of constructed plasmids pNc_Cas9CRISPR::sgNcMIC26 and pLIC-HA-DHFR-NcMIC26. [file Data_Sheet_1.docx]

| Plasmid | Primers | Sequence |
| --- | --- | --- |
| pNc_Cas9CRISPR::sgNcMIC26 | MIC26-U6-gRNA-F | TCAGGTTTTATTAGGAGTCTGTTTTAGAGCTAGAAATAG |
|  | MIC26-U6-gRNA-R | AGACTCCTAATAAAACCTGAAAACAACAATGTCCCTTTG |
|  | Cas9-F | ATACGACTCACTATAGGGCG |
|  | Cas9-R | AGCTCCACCGCGGTGGCGGC |
| pLIC-HA-DHFR-NcMIC26 | MIC26-5'FLANK-F | TCCAATTTAATTAAGATATCGTCATCGAGACAGTTGAGT |
|  | MIC26-5'FLANK-R | ACGTCGTACGGGTACCTAGGCGCCGACCACATTTCGGAG |
|  | MIC26-3'FLANK-F | GGGGGATCGATCCACTAGTCGGGGAATGCAGCTCCAGTG |
|  | MIC26-3'FLANK-R | GCGGTGGCGGCCGCTCTAGATCACCTGCCATTTACACAC |
|  | HA-DHFR-F | CCTAGGTACCCGTACGACGT |
|  | HA-DHFR-R | ACTAGTGGATCGATCCCCCG |
|  | pLIC-F | TCTAGAGCGGCCGCCACCGC |
|  | pLIC-R | GATATCTTAATTAAATTGGA |

**Table S1 Primers of constructed plasmids pNc_Cas9CRISPR::sgNcMIC26 and pLIC-HA-DHFR-NcMIC26**

**Table S2 The data of tested samples for indirect ELISA based on the rNcMIC26 and rNcSRS2**

| Ninety-eight samples of *N. caninum*-positive defined by IFAT | | | | | | | | | | | | |
| --- | --- | --- | --- | --- | --- | --- | --- | --- | --- | --- | --- | --- |
| Sample | 1 | 2 | 3 | 4 | 5 | 6 | 7 | 8 | 9 | 10 | 11 | 12 |
| SRS2 | 0.612 | 0.319 | 0.255 | 0.087 | 0.705 | 0.526 | 0.927 | 0.381 | 1.23 | 0.062 | 0.522 | 0.596 |
| MIC26 | 0.69 | 0.56 | 0.106 | 0.322 | 0.497 | 0.309 | 0.751 | 0.035 | 0.795 | 0.141 | 0.838 | 0.258 |
|  |  |  |  |  |  |  |  |  |  |  |  |  |
| Sample | 13 | 14 | 15 | 16 | 17 | 18 | 19 | 20 | 21 | 22 | 23 | 24 |
| SRS2 | 0.499 | 1.021 | 0.096 | 0.667 | 0.655 | 0.658 | 0.673 | 0.447 | 1.097 | 0.893 | 0.042 | 0.906 |
| MIC26 | 0.625 | 0.316 | 0.307 | 0.895 | 0.37 | 0.587 | 0.289 | 0.146 | 0.237 | 0.24 | 0.137 | 0.543 |
|  |  |  |  |  |  |  |  |  |  |  |  |  |
| Sample | 25 | 26 | 27 | 28 | 29 | 30 | 31 | 32 | 33 | 34 | 35 | 36 |
| SRS2 | 0.942 | 0.762 | 0.28 | 0.98 | 0.086 | 0.307 | 0.763 | 0.245 | 0.405 | 0.753 | 0.673 | 0.591 |
| MIC26 | 0.638 | 0.43 | 0.303 | 0.691 | 0.497 | 0.393 | 0.322 | 0.092 | 0.594 | 0.033 | 0.378 | 0.254 |
|  |  |  |  |  |  |  |  |  |  |  |  |  |
| Sample | 37 | 38 | 39 | 40 | 41 | 42 | 43 | 44 | 45 | 46 | 47 | 48 |
| SRS2 | 1.053 | 0.113 | 0.782 | 0.287 | 0.287 | 0.901 | 0.577 | 0.994 | 0.505 | 0.27 | 0.627 | 0.585 |
| MIC26 | 0.529 | 0.262 | 0.837 | 0.265 | 0.113 | 0.476 | 0.145 | 0.482 | 0.679 | 0.73 | 0.551 | 0.105 |
|  |  |  |  |  |  |  |  |  |  |  |  |  |
| Sample | 49 | 50 | 51 | 52 | 53 | 54 | 55 | 56 | 57 | 58 | 59 | 60 |
| SRS2 | 0.456 | 0.366 | 0.311 | 0.059 | 0.876 | 0.484 | 1.18 | 0.292 | 0.124 | 0.105 | 0.121 | 1.293 |
| MIC26 | 0.136 | 0.424 | 0.219 | 0.209 | 0.351 | 0.129 | 0.031 | 0.275 | 0.433 | 0.321 | 0.537 | 0.887 |
|  |  |  |  |  |  |  |  |  |  |  |  |  |
| Sample | 61 | 62 | 63 | 64 | 65 | 66 | 67 | 68 | 69 | 70 | 71 | 72 |
| SRS2 | 0.074 | 0.085 | 0.157 | 0.099 | 0.121 | 0.137 | 0.487 | 0.109 | 0.505 | 0.129 | 0.155 | 0.258 |
| MIC26 | 0.606 | 0.359 | 0.698 | 0.327 | 0.357 | 0.646 | 0.552 | 0.393 | 0.894 | 0.577 | 0.383 | 0.635 |
|  |  |  |  |  |  |  |  |  |  |  |  |  |
| Sample | 73 | 74 | 75 | 76 | 77 | 78 | 79 | 80 | 81 | 82 | 83 | 84 |
| SRS2 | 0.3 | 0.283 | 0.138 | 0.916 | 0.086 | 0.061 | 0.289 | 0.075 | 0.144 | 0.85 | 0.11 | 0.034 |
| MIC26 | 0.442 | 1.053 | 0.782 | 0.433 | 0.124 | 0.383 | 0.133 | 0.383 | 0.337 | 0.107 | 0.316 | 0.122 |
|  |  |  |  |  |  |  |  |  |  |  |  |  |
| Sample | 85 | 86 | 87 | 88 | 89 | 90 | 91 | 92 | 93 | 94 | 95 | 96 |
| SRS2 | 0.353 | 0.052 | 0.078 | 0.174 | 0.145 | 0.282 | 0.102 | 0.203 | 0.226 | 0.105 | 0.759 | 0.442 |
| MIC26 | 0.788 | 0.085 | 0.452 | 0.37 | 0.141 | 0.142 | 0.53 | 0.351 | 0.773 | 0.435 | 0.13 | 0.148 |
|  |  |  |  |  |  |  |  |  |  |  |  |  |
| Sample | 97 | 98 |  |  |  |  |  |  |  |  |  |  |
| SRS2 | 0.249 | 0.203 |  |  |  |  |  |  |  |  |  |  |
| MIC26 | 0.112 | 0.351 |  |  |  |  |  |  |  |  |  |  |
|  |  |  |  |  |  |  |  |  |  |  |  |  |
| Thirty-nine samples of *N. caninum*-negative defined by IFAT | | | | | | | | | | | | |
| Sample | 1 | 2 | 3 | 4 | 5 | 6 | 7 | 8 | 9 | 10 | 11 | 12 |
| SRS2 | 0.179 | 0.098 | 0.087 | 0.051 | 0.006 | 0.04 | 0.044 | 0.003 | 0.291 | 0.035 | 0.002 | 0.03 |
| MIC26 | 0.222 | 0.13 | 0.089 | 0.187 | 0.01 | 0.062 | 0.088 | 0.007 | 0.206 | 0.047 | 0.064 | 0.041 |
|  |  |  |  |  |  |  |  |  |  |  |  |  |
| Sample | 13 | 14 | 15 | 16 | 17 | 18 | 19 | 20 | 21 | 22 | 23 | 24 |
| SRS2 | 0.112 | 0.096 | 0.047 | 0.096 | 0.005 | 0.07 | 0.024 | 0.005 | 0.01 | 0.021 | 0.013 | 0.005 |
| MIC26 | 0.162 | 0.255 | 0.055 | 0.097 | 0.019 | 0.063 | 0.039 | 0.068 | 0.102 | 0.052 | 0.028 | 0.021 |
|  |  |  |  |  |  |  |  |  |  |  |  |  |
| Sample | 25 | 26 | 27 | 28 | 29 | 30 | 31 | 32 | 33 | 34 | 35 | 36 |
| SRS2 | 0.195 | 0.14 | 0.058 | 0.046 | 0.016 | 0.005 | 0.012 | 0.04 | 0.11 | 0.1 | 0.026 | 0.027 |
| MIC26 | 0.195 | 0.1 | 0.065 | 0.07 | 0.098 | 0.011 | 0.037 | 0.029 | 0.042 | 0.064 | 0.081 | 0.014 |
|  |  |  |  |  |  |  |  |  |  |  |  |  |
| Sample | 37 | 38 | 39 |  |  |  |  |  |  |  |  |  |
| SRS2 | 0.126 | 0.096 | 0.031 |  |  |  |  |  |  |  |  |  |
| MIC26 | 0.093 | 0.109 | 0.128 |  |  |  |  |  |  |  |  |  |

Yellow highlights represent *N. caninum*-positive defined by ELISA based on NcSRS2; Green highlights represent *N. caninum*-positive defined by ELISA based on NcMIC26.
